# Plerixafor as a chemosensitizing agent in pediatric acute lymphoblastic leukemia: efficacy and potential mechanisms of resistance to CXCR4 inhibition

**DOI:** 10.18632/oncotarget.2407

**Published:** 2014-08-29

**Authors:** Edward Allan R. Sison, Daniel Magoon, Li Li, Colleen E. Annesley, Rachel E. Rau, Donald Small, Patrick Brown

**Affiliations:** ^1^ Pediatric Hematology/Oncology, Texas Children's Cancer and Hematology Centers, Baylor College of Medicine, Houston, TX, USA; ^2^ Oncology and Pediatrics, The Sidney Kimmel Comprehensive Cancer Center, Johns Hopkins University School of Medicine, Baltimore, MD, USA

**Keywords:** acute lymphoblastic leukemia, pediatric, CXCR4, microenvironment, plerixafor, AMD3100

## Abstract

In spite of advances in the treatment of pediatric acute lymphoblastic leukemia (ALL), a significant number of children with ALL are not cured of their disease. We and others have shown that signaling from the bone marrow microenvironment confers therapeutic resistance, and that the interaction between CXCR4 and stromal cell-derived factor-1 (SDF-1 or CXCL12) is a key mediator of this effect. We demonstrate that ALL cells that upregulate surface CXCR4 in response to chemotherapy treatment are protected from chemotherapy-induced apoptosis when co-cultured with bone marrow stroma. Treatment with the CXCR4 inhibitor plerixafor diminishes stromal protection and confers chemosensitivity. Using xenograft models of high-risk pediatric ALL, plerixafor plus chemotherapy induces significantly decreased leukemic burden, compared to chemotherapy alone. Further, treatment with plerixafor and chemotherapy influences surface expression of CXCR4, VLA-4, and CXCR7 in surviving ALL blasts. Finally, prolonged exposure of ALL blasts to plerixafor leads to a persistent increase in surface CXCR4 expression, along with modulation of surface expression of additional adhesion molecules, and enhanced SDF-1α-induced chemotaxis, findings that may have implications for therapeutic resistance. Our results suggest that while CXCR4 inhibition may prove useful in ALL, further study is needed to understand the full effects of targeting the leukemic microenvironment.

## INTRODUCTION

Acute lymphoblastic leukemia (ALL) is the most common childhood cancer. Due to advances in diagnosis and treatment, over 80% of children with ALL are cured with contemporary treatment regimens. However, a significant proportion of pediatric ALL recur due to chemotherapy resistance. We and others have demonstrated that signaling between leukemia cells and stromal cells in the bone marrow microenvironment contributes to leukemia cell growth and survival, and that co-culture with normal human bone marrow stroma mediates therapeutic resistance.[1-10] The association between the cell surface receptor CXCR4 and the chemokine SDF-1 is thought to be an essential component of these interactions. CXCR4 is commonly expressed on the surface of ALL and acute myeloid leukemia (AML) cells.[11] Similar to its role in hematopoietic stem cell (HSC) regulation, activation of CXCR4 by SDF-1 is critical for the migration and retention of leukemia cells within the bone marrow and may play a role in extramedullary spread.[12] Because the SDF-1/CXCR4 axis is important in attracting and retaining leukemia cells within the bone marrow microenvironment, CXCR4 inhibition could allow leukemia cells to be released from protective niches that contribute to chemotherapy resistance. Plerixafor (Mozobil, formerly AMD3100) is a reversible inhibitor of CXCR4 that is approved by the United States Food and Drug Administration for use as an HSC mobilizing agent. Preclinical studies[3,6-10,13,14] and clinical trials[15-17] using plerixafor have suggested that CXCR4 inhibition is an effective means to enhance sensitivity to anti-leukemic therapies and mobilize leukemic blasts.

Therapy resistance in high-risk, relapsed, and/or refractory pediatric ALL may be partly mediated by increased interaction between residual leukemic blasts and the bone marrow microenvironment. Therefore, interruption of these interactions through CXCR4 antagonism could augment the effects of chemotherapy and improve overall outcome in these children. In this study, we demonstrate that chemotherapy-induced upregulation of surface CXCR4 expression in surviving leukemic blasts is a mechanism of therapeutic resistance in ALL. We also show that this therapeutic resistance can be reversed with plerixafor. Using a xenograft model of high-risk pediatric ALL, we also demonstrate that CXCR4 inhibition with plerixafor enhances sensitivity to chemotherapy. We also show that *in vivo* treatment with chemotherapy and plerixafor leads to modulation of surface expression of CXCR4 and other adhesion molecules in surviving leukemic blasts. Finally, we offer evidence that prolonged inhibition of CXCR4 leads to an increase in surface CXCR4 expression as well as modulation of additional adhesion pathways, suggesting a mechanism of resistance to CXCR4 inhibition.

## RESULTS

### Chemotherapy-induced upregulation of surface CXCR4 is a mechanism of chemotherapy resistance in ALL cell lines that can be reversed with plerixafor

**Figure 1:**
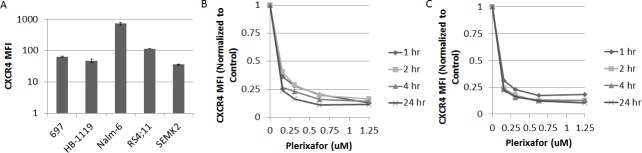
Plerixafor decreases surface CXCR4 expression as measured by anti CXCR4 antibody binding (A) Baseline surface CXCR4 expression as measured by flow cytometry using the 12G5 anti-CXCR4 antibody. Surface CXCR4 as measured by flow cytometry using the 12G5 antibody after treatment of (B) Nalm-6 and (C) SEMK2 with plerixafor over a time course. Error bars in this and subsequent figures represent the standard error of the mean (SEM).

**Figure 2:**
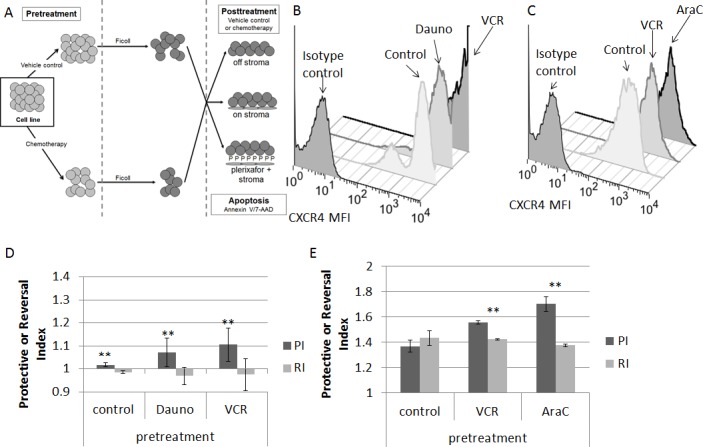
Chemotherapy-induced upregulation of surface CXCR4 leads to stromal protection and chemoresistance; treatment with plerixafor decreases stromal protection and chemoresistance (A) Treatment schema: cells were pretreated with chemotherapy or vehicle control, then treated with additional chemotherapy for 72 hours either off stroma, on normal human bone marrow stroma, or on stroma with plerixafor. Following all treatment, apoptosis was measured by Annexin V and 7-AAD staining. Surface CXCR4 expression in (B) Nalm-6 and (C) RS4;11 after pretreatment. Protective Index (PI) and Reversal Index (RI) after posttreatment in (D) Nalm-6 and (E) RS4;11. AraC, cytarabine; Dauno, daunorubicin; VCR, vincristine. **p<0.01 PI vs. RI.

We first measured baseline surface expression of CXCR4 in five ALL cell lines. We found that all cell lines expressed surface CXCR4 and that expression varied between cell lines (Fig. 1A). Next, we treated the cell lines with the highest and lowest surface expression of CXCR4 with a dose range of plerixafor over a 24 hour time course to determine the potency, onset, and duration of CXCR4 inhibition. To assess the ability of plerixafor to inhibit surface CXCR4, we stained cells with the 12G5 clone of the anti-CXCR4 antibody, which attaches to the SDF-1 and drug-binding site of CXCR4. In spite of variations in baseline surface CXCR4 expression, the ability of plerixafor to inhibit 12G5 antibody binding was consistent across cell lines, with dose-dependent inhibition of 12G5 antibody binding starting at 1 hour that was maintained through 24 hours (Figs. 1B-1C). We also found that plerixafor was able to inhibit 12G5 antibody binding in the remaining 3 cell lines (Supplemental Figs. 1A-1C), suggesting that plerixafor can inhibit CXCR4 effectively at various levels of baseline surface CXCR4 expression. Next, we wanted to model a treatment-refractory or residual disease state by treating ALL cell lines with non-lethal doses of chemotherapy and determining if the surviving cells show increased interactions with the bone marrow microenvironment. We chose Nalm-6 and RS4;11, which had the highest baseline surface CXCR4 expression, to investigate our hypothesis. Our treatment schema is shown in Fig. 2A. Pretreatment with chemotherapy led to an increase in surface CXCR4 expression in surviving cells, compared to pretreatment with vehicle control (Figs. 2B-2C). Next, we exposed the pretreated cells to dose ranges of chemotherapy in 3 culture conditions: 1) off stroma, 2) on stroma, or 3) treated with plerixafor and then plated on stroma (Fig. 2A). After treatment, we measured apoptosis and calculated inhibitory concentration values (IC10 through IC90). Using the IC values, we calculated a Protective Index (PI) and a Reversal Index (RI). We defined the PI as the IC values on stroma divided by the IC values off stroma; therefore, PI >1 indicated stromal protection.[10] For the RI, we divided the IC values of the plerixafor + stroma condition by the IC values off stroma; thus, RI < PI indicated a decrease in stromal protection. Stroma protected control-pretreated Nalm-6 (Fig. 2D) and RS4;11 (Fig. 2E) from chemotherapy-induced apoptosis. Notably, stroma differentially protected chemotherapy-pretreated cells from additional chemotherapy-induced apoptosis, suggesting that chemotherapy-induced upregulation of surface CXCR4 led to higher protective indices. Further, plerixafor preferentially decreased stromal protection to a greater degree in chemotherapy-pretreated cells, compared to control-pretreated cells (Figs. 2D-2E), suggesting that the degree of surface CXCR4 upregulation potentiates the ability of plerixafor to reverse stromal protection. Our findings suggest that chemotherapy exposure induces an increase in stromal protection that is at least in part mediated by CXCR4.

### Plerixafor enhances sensitivity to chemotherapy in xenografts of infant MLL-rearranged (*MLL-R*) ALL

**Figure 3:**
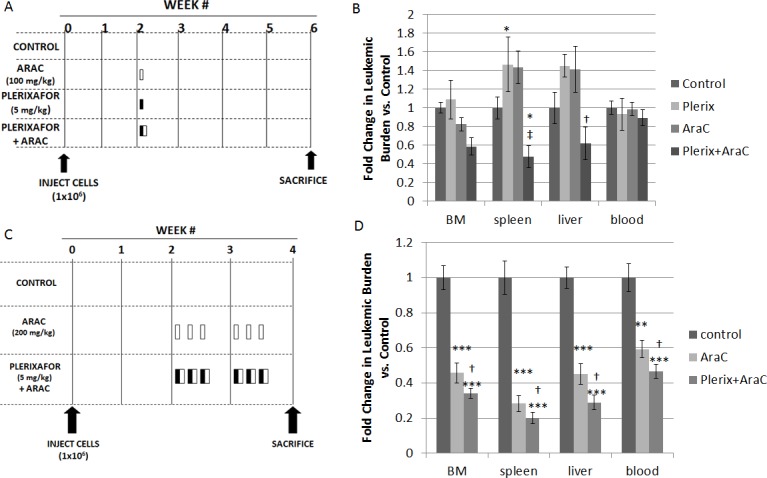
*In vivo* treatment with plerixafor sensitizes leukemic blasts to AraC (A) Treatment strategy A. AraC, plerixafor, or vehicle control (phosphate-buffered saline) was administered in a single dose at week 2: AraC was administered via intraperitoneal injection, plerixafor was administered via subcutaneous injection, and PBS was administered via both routes. (B) Leukemic burden in the bone marrow, spleen, liver, and blood: the results of 2 primary sample experiments were pooled. To account for variability in leukemic engraftment between samples, leukemic burden was quantified as the absolute number of cells co-expressing human CD45 and human CD19 and normalized to the average leukemic burden in control mice of each primary sample. (C) Treatment strategy B. AraC, plerixafor and AraC, or vehicle control was administered once daily on three consecutive days for two weeks, starting at week 2: AraC was administered via intraperitoneal injection, plerixafor was administered via subcutaneous injection, and PBS was administered via both routes. (D) Leukemic burden was quantified using the methods described above. The results of 4 primary sample experiments were pooled. BM, bone marrow. *p<0.05, **p<0.01, ***p<0.001 vs. control. ^†^p<0.05, ^‡^p<0.01 vs AraC.

We next sought to determine if plerixafor could sensitize primary samples of high-risk ALL to chemotherapy in an *in vivo* NSG xenograft model (Table 1 for sample characteristics). We used primary samples of infant *MLL-*R ALL because these patients have a very poor outcome, and these samples readily engraft in immunodeficient mice and demonstrate enhanced survival with stromal co-culture.[9] After a two-week period of engraftment, we treated the mice with vehicle control, plerixafor, or cytarabine using two different dosing schedules. We chose cytarabine because of its efficacy against infant ALL.[18] In dosing schedule A, we administered single doses of vehicle control, plerixafor (5 mg/kg), cytarabine alone (100 mg/kg), or plerixafor followed four hours later by cytarabine in order to allow maximum interruption of leukemia-stromal interactions (Fig. 3A). Mice were sacrificed 4 weeks after treatment and cells were isolated from bone marrow, spleen, liver, and peripheral blood and analyzed by flow cytometry. Overall, leukemic burden in the bone marrow was similar in control-, cytarabine-, and plerixafor-treated mice, consistent with conservative dosing of cytarabine and minimal direct anti-leukemic effect of plerixafor (Fig. 3B). Interestingly, both plerixafor alone and cytarabine alone induced increased leukemic burden in the spleen compared to control-treated mice. Further, this treatment strategy showed minimal effects on circulating leukemic blasts. However, even with the use of a modest dose of cytarabine, the combination of plerixafor and cytarabine resulted in significantly decreased leukemic burden in the spleen and liver, as well as a trend toward decreased leukemic burden in the bone marrow, compared to cytarabine alone.

**Table 1:** Characteristics of patient samples for xenograft experiments

sample	age at diagnosis	MLL abnormality
MLL1	3 months	t(4;11)(q21;q23)
MLL2	4 months	t(4;11)(q21;q23)
MLL3	4 months	t(11;19)(q23;p13.3)
MLL4	birth	t(11;19)(q23;p13.3)

In dosing strategy B, we increased the dose of cytarabine and intensified the overall therapy by treating on three consecutive days for two weeks (Fig. 3C). Given that plerixafor did not significantly affect leukemic burden in the bone marrow in these experiments and our prior experiments,[9] we used three treatment cohorts for these experiments: vehicle control, cytarabine alone (200 mg/kg), and plerixafor (5 mg/kg) followed by cytarabine four hours later (Fig. 3C). This treatment strategy was much more effective, as cytarabine significantly decreased leukemic burden in the bone marrow, spleen, liver, and peripheral blood, compared to vehicle control. Notably, the combination of plerixafor and cytarabine significantly reduced leukemic burden, compared to both vehicle control and to cytarabine alone (Fig. 3D), suggesting that plerixafor enhanced sensitivity to cytarabine in this model. The efficacy of this treatment strategy was variable by patient sample but, in general, each sample showed increased efficacy of plerixafor and cytarabine (Supplemental Figs. 2A-2D). Histopathologic examination of the bone marrow and spleen also demonstrated restoration of normal hematopoietic elements and normalization of splenic architecture in mice treated with plerixafor and cytarabine (Supplemental Fig. 2E). From these experiments, we concluded that CXCR4 inhibition may be an effective strategy to increase chemosensitivity in infant *MLL*-R ALL.

### Treatment with plerixafor and cytarabine modulates surface expression of adhesion molecules *in vivo* in residual ALL blasts

**Figure 4:**
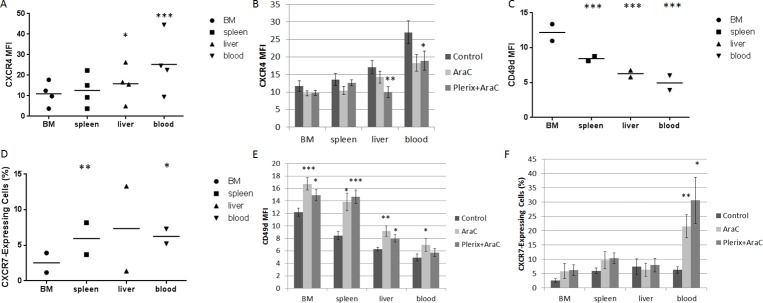
*In vivo* CXCR4 expression and other chemokine pathways Results detailed in these figures are from mice used in the experiment in Figure 3C. (A) Surface CXCR4 expression as measured by flow cytometry in the leukemic blast populations of control mice (n=4 primary samples). (B) Surface CXCR4 expression in leukemic blasts by treatment cohort and organ across all primary samples. (C) Surface CD49d expression as measured by flow cytometry in the leukemic blast populations of control-treated mice (n=2 primary samples using treatment strategy B). (D) Surface CXCR7 expression as measured by flow cytometry in the leukemic blast populations of control-treated mice (n=2 primary samples using treatment strategy B). Due to low CXCR7 MFI levels, CXCR7-expressing cells were defined as those having MFI greater than the 95^th^ percentile of the isotype control MFI. (E) Surface CD49d expression in leukemic blasts by treatment cohort and organ across all primary samples. (F) CXCR7-expressing cells in the leukemic blast population by treatment cohort and organ across all primary samples. BM, bone marrow. *p<0.05, **p<0.01, ***p<0.001 vs. bone marrow (A, C, and D) or vs. control (B, E, and F).

We wanted to verify our *in vitro* findings that chemotherapy modulated surface CXCR4 expression in our *in vivo* model. Therefore, we measured surface CXCR4 expression in leukemic blasts at the time of sacrifice. In all primary samples, surface CXCR4 expression in control-treated mice was highest in leukemic blasts harvested from the peripheral blood, followed by liver, spleen, and bone marrow (Fig. 4A). Surface CXCR4 expression of control-treated cells did not appear to correlate with engraftment or response to therapy. However, surface CXCR4 expression was significantly modulated by cytarabine treatment with or without plerixafor (Fig. 4B), suggesting that anti-leukemic therapies may affect surface expression of CXCR4 in surviving leukemic blasts *in vivo*. Specifically, we observed consistent downregulation of CXCR4 by cytarabine treatment in blasts taken from the liver and blood; this pattern was not significant in blasts isolated from the bone marrow or spleen. Given these findings, we performed an extended analysis on two of the primary samples to determine if we could observe patterns of expression of other adhesion molecules. Specifically, we measured surface expression of CD49d and CXCR7 by flow cytometry. CD49d is the integrin alpha subunit of VLA-4, which is an integrin that binds to fibronectin and VCAM-1 among other ligands in the bone marrow microenvironment.[19,20] CXCR7 is the second receptor for SDF-1 and is also a receptor for the chemokine CXCL11.[21] We found that CD49d surface expression was highest in leukemic blasts residing in the bone marrow, followed by spleen, liver, and peripheral blood, which was the opposite pattern of CXCR4 (Fig. 4C). In contrast, surface expression of CXCR7 was lowest in leukemic blasts isolated from the bone marrow and relatively similar among the other organs (Fig. 4D). Interestingly, treatment with cytarabine with or without plerixafor led to statistically significant increases in both CD49d (Fig. 4E) and CXCR7 surface expression (Fig. 4F). In summary, these findings suggest that anti-leukemic therapies affect surface expression of multiple adhesion molecules in surviving leukemic blasts, perhaps as a compensatory response to CXCR4 inhibition.

### Extended treatment with plerixafor induces increased surface expression of adhesion molecules in ALL

**Figure 5:**
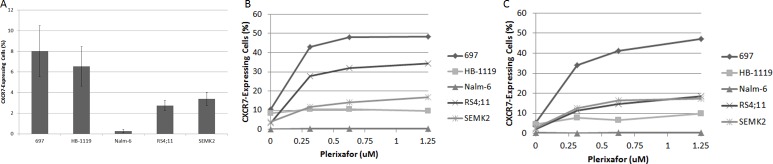
Plerixafor modulates surface CXCR7 expression (A) Baseline surface CXCR7 expression as measured by flow cytometry. Surface CXCR7 expression after treatment with plerixafor for (B) 4 hours and (C) 24 hours.

While plerixafor enhanced chemosensitivity in our xenografts, the combination of plerixafor and cytarabine did not eliminate the leukemia in our model. Because we observed that *in vivo* treatment with plerixafor and cytarabine led to modulation of surface CXCR4, CD49d, and CXCR7 expression in residual leukemic blasts, we hypothesized that exposure to plerixafor may have led to increased interactions between surviving leukemic blasts and the bone marrow microenvironment. To investigate this, we treated pre-B ALL cell lines with a dose range of plerixafor over a brief period of time and measured surface expression of CD49d and CXCR7 by flow cytometry. CD49d was highly expressed at baseline (Supplemental Fig. 3A), while CXCR7 was expressed to a lesser degree (Fig. 5A). Treatment with plerixafor led to no significant modulation of CD49d surface expression (Supplemental Figs. 3B-3C). However, plerixafor treatment consistently led to increased surface CXCR7 expression across cell lines, suggesting that inhibition of CXCR4 induces increased surface expression of the second receptor of SDF-1 (Figs. 5B-5C).

**Figure 6:**
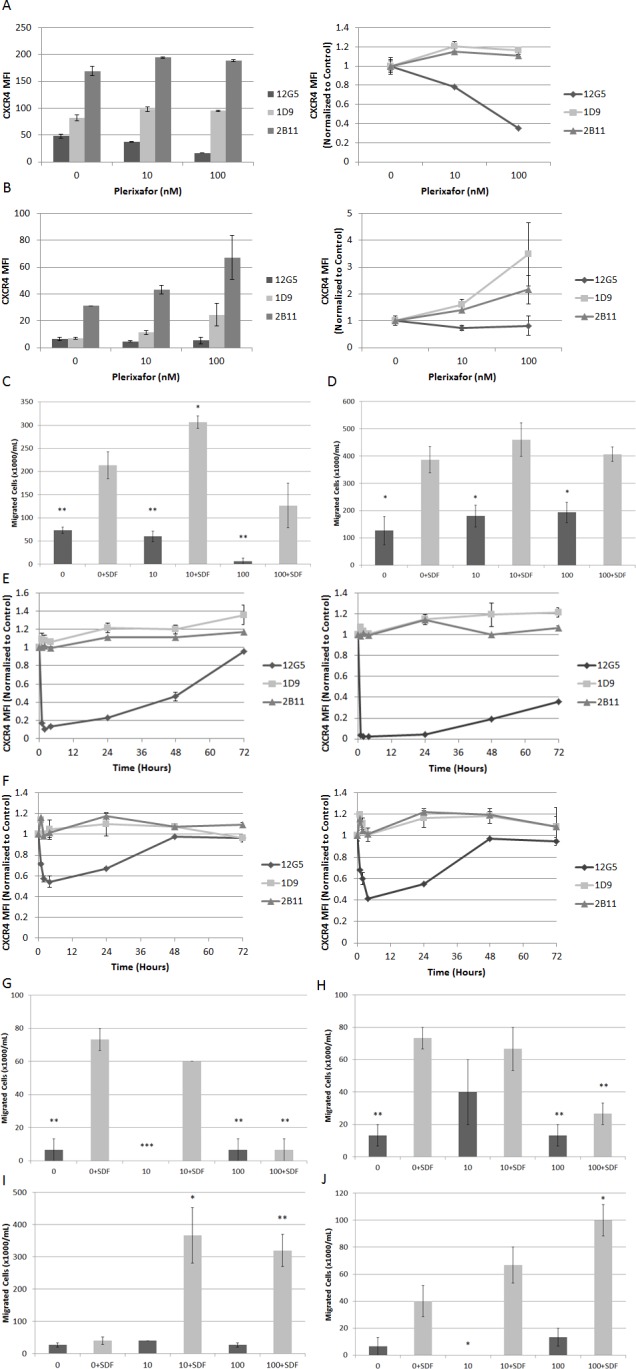
Extended treatment with plerixafor modulates surface expression of surface CXCR4 and affects SDF-1α-induced chemotaxis Cell lines were treated with plerixafor (10 nM, 100 nM) or vehicle control (0 nM) for 72 hours and analyzed by flow cytometry and chemotaxis assays. Surface expression of CXCR4 using 12G5, 1D9, and 2B11 antibodies in (A) Nalm-6 and (B) HB-1119. Cells were then washed and resuspended in fresh medium. Chemotaxis toward medium with or without recombinant SDF-1α (150 ng/mL) after 24 hours in (C) Nalm-6 and (D) HB-1119. In separate experiments, Nalm-6 and HB-1119 were treated with plerixafor or vehicle control for 72 hours and cells were harvested at multiple time points for flow cytometry and chemotaxis. Surface expression of CXCR4 measured at multiple time points using 12G5, 1D9, and 2B11 antibodies in (E) Nalm-6 and (F) HB-1119. Cells treated with plerixafor or vehicle control for 4 hours were washed and resuspended in fresh medium. Chemotaxis toward medium with or without recombinant SDF-1α (150 ng/mL) after 1 hour in (G) Nalm-6 and (H) HB-1119, after 24 hours in (I) Nalm-6 and (J) HB-1119. *p<0.05, **p<0.01, ***p<0.001 vs. 0+SDF.

Next, to determine the effects of extended exposure to plerixafor and subsequent withdrawal, we treated ALL cell lines with plerixafor for 72 hours. We measured surface expression of surface CXCR4 using three different antibodies: 12G5, which attaches to the SDF-1 and drug-binding site of CXCR4, and 1D9 and 2B11, which do not compete with SDF-1 or drug binding. In pilot experiments, we verified that the 2B11 antibody bound to human CXCR4 as previously reported (data not shown).[22] Immediately after 72 hours of treatment with plerixafor, 12G5 binding was inhibited in a dose-dependent manner as expected. Surprisingly, 1D9 and 2B11 binding were increased in an inversely proportional manner to 12G5 binding, suggesting not only that plerixafor caused an actual increase in surface CXCR4 over time but that the increase in CXCR4 was directly related to the degree of CXCR4 inhibition (Figs. 6A-6B). We confirmed these increases in surface CXCR4 by qRT-PCR (Supplemental Figs. 4A-4B). Measurement of VLA-4 and CXCR7 by flow cytometry and qRT-PCR also showed modulation after 72 hours of treatment with plerixafor (Supplemental Figs. 4C-4J).

Next, we washed the cells, resuspended them in fresh medium without plerixafor, and analyzed aliquots of cells from each treatment condition for an additional 72 hours to determine if these changes in surface CXCR4 expression persisted and if they led to functional consequences. After withdrawal of plerixafor, 12G5 binding increased to untreated levels between 4 and 24 hours, while 1D9 and 2B11 binding decreased to untreated levels between 4 and 72 hours (Supplemental Figs. 5A-5D). To determine if these observed increases in surface CXCR4 expression were functional, we measured migration of washed cells from each treatment condition through a permeable membrane toward medium containing SDF-1α or medium alone. Despite CXCR4 inhibition for 72 hours, all plerixafor-treated cells migrated in response to SDF-1α. In addition, some plerixafor-treated cells exhibited increased SDF-1α-induced chemotaxis compared to control-treated cells (Figs. 6C-6D). These findings imply that increases in surface CXCR4 induced by 72 hours of treatment with plerixafor are functional. Our results are in stark contrast to a previous report showing that short-term treatment with plerixafor results decreased SDF-1α-induced chemotaxis.[3] Therefore, we hypothesized that there is a therapeutic window for the use of plerixafor, during which an overall increase in surface CXCR4 expression is balanced by inhibition at the SDF-1 binding site. To investigate this, we treated Nalm-6 and HB-1119 with plerixafor for 72 hours and collected cells at multiple time points for flow cytometry and chemotaxis. We found a rapid and potent inhibition of 12G5 antibody binding that was paralleled by an increase in 1D9 and 2B11 antibody binding. However, over time, inhibition of 12G5 antibody binding waned while increases in 1D9 and 2B11 binding persisted (Figs. 6E-6F). We were able to demonstrate that these differences in 12G5 antibody binding were functional in short chemotaxis assays (Figs. 6G-6H), but that a 24-hour chemotaxis assay had an opposite effect (Figs. 6I-6J), likely due to a washout of CXCR4 inhibition and an overall increase in surface CXCR4. In summary, our data demonstrate that plerixafor is an effective inhibitor of CXCR4 in pediatric ALL and that the timing of administration may be critical to optimizing its use as a chemosensitizing agent in leukemia.

## DISCUSSION

High level surface expression of CXCR4 has been associated with inferior outcome in pediatric ALL.[23,24] In this study, we offer further evidence that CXCR4 is an important functional determinant of treatment response in pediatric ALL. We and others have previously shown that anti-leukemic agents can modulate CXCR4 expression in ALL,[10,25] AML,[10] chronic lymphocytic leukemia,[26] and chronic myelogenous leukemia.[27] Therefore, initial levels and changes in levels of CXCR4 in leukemia cells may affect the protection conferred by bone marrow niches. In addition, we have previously demonstrated that AML cases that upregulate surface CXCR4 in response to chemotherapy are differentially protected from chemotherapy-induced apoptosis when grown in co-culture with normal bone marrow stroma. In this study, we hypothesized that chemotherapy-induced increases in surface CXCR4 expression may mediate chemotherapy resistance in ALL. Interestingly, cells with higher levels of surface CXCR4 upregulation had higher Protective Indices compared to cells with lower surface CXCR4 upregulation, suggesting that the degree of surface CXCR4 upregulation is predictive of the degree of stromal protection. Cells with higher surface CXCR4 upregulation also had greater differences between the Protective and Reversal Index, suggesting that plerixafor diminishes stromal protection more effectively in leukemias that highly upregulate surface CXCR4 in response to chemotherapy. These findings are in concert with our previous findings in AML.[10]

We had previously shown that treatment with plerixafor renders infant *MLL-*R leukemic blasts more susceptible to the FLT3 inhibitor lestaurtinib in a xenograft model.[9] To increase the applicability of this treatment strategy in this difficult-to-treat population, we combined a standard chemotherapeutic agent with plerixafor in a xenograft model of infant *MLL-*R ALL. Our first treatment strategy was minimally effective, likely due to low intensity of the treatment regimen. Our second treatment strategy, however, was much more effective and the combination of plerixafor and cytarabine demonstrated a significant improvement in leukemic control compared to cytarabine alone, suggesting that inhibition of the bone marrow microenvironment may be a means to improve upon the poor overall and disease-free survival in infant *MLL*-R ALL. Using the xenograft model, we measured surface expression of CXCR4, VLA-4 (CD49d), and CXCR7 in surviving leukemic blasts to learn more about the potential for surviving blasts to interact with the bone marrow microenvironment.

Surface CXCR4 expression did not correlate with response to therapy in our xenograft model. This may be due to a small sample size and we plan to explore the role of CXCR4 expression in clinical outcome in a larger cohort of patient samples in future experiments. Interestingly, surface expression of CXCR4 and CD49d in leukemic blasts appeared to be in an inverse relationship. Specifically, CXCR4 expression was highest in leukemia cells circulating in the peripheral blood and lowest in those localized to the bone marrow, while CD49d expression was highest in the bone marrow and lowest in the peripheral blood. Downregulation of a receptor often occurs when the ligand is in excess. Thus, it follows that CXCR4 is lowest where the concentration of SDF-1α is the highest (i.e., the bone marrow). In addition, it is possible that blasts migrating out of the marrow upregulate surface CXCR4 as they home to new niches. Previous studies have also suggested that the functions of CXCR4 and VLA-4 are linked. For example, treatment of pre-B ALL cell lines or primary samples with SDF-1α, which causes activation and internalization of CXCR4, resulted in increased adhesion to fibronectin, laminin, and VCAM-1, suggesting that SDF-1α and CXCR4 influence the functionality of VLA-4.[28] Another study showed that treatment of neutrophils with SDF-1α also led to increased adhesion to VCAM-1.[29] Therefore, it is possible that the pattern in organ-specific surface expression of CXCR4 and CD49d in control-treated mice is part of a homeostatic relationship. Further, high concentrations of SDF-1α in the bone marrow may have led to activation and ligand-receptor-mediated internalization of CXCR4, which resulted in lower surface CXCR4 expression as well as activation and increased surface expression of CD49d. Evidence of the importance of VLA-4 in ALL is shown in a study demonstrating that treatment of pre-B ALL cell lines and primary samples with antibodies against VLA-4 led to significantly impaired bone marrow homing in a transplant model using NOD/SCID mice.[30] We also observed that our treatment led to increases in surface expression of CD49d and CXCR7. Upregulation of surface CD49d expression in surviving blasts may be a compensatory mechanism to overcome CXCR4 inhibition. Similarly, surviving blasts may have increased CXCR7 surface expression to circumvent CXCR4 antagonism and to provide additional cell surface receptors to respond to SDF-1 in the bone marrow microenvironment. CXCR7 is a scavenger of SDF-1 and participates in SDF-1-mediated signaling and chemotaxis in concert with CXCR4.[21,31] Therefore, an increase in surface CXCR7 in residual leukemic blasts may lead to increased interactions with the bone marrow microenvironment. More study is needed to fully understand these phenomena.

We investigated these ideas by treating ALL cells with plerixafor alone at various doses and periods of time. Short-term treatment with plerixafor induced an increase in surface CXCR7 expression, while extended treatment led to increased CD49d expression. Remarkably, prolonged exposure to plerixafor led to an overall increase in surface CXCR4 expression, as measured by 1D9 and 2B11 antibody binding, with a simultaneous dose-dependent decrease in 12G5 antibody binding, which measures blockade of the SDF-1 binding site on CXCR4. Further, increases in surface CXCR4 expression persisted for up to 72 hours after withdrawal from plerixafor and plerixafor-treated cells demonstrated continued and, in some cases, enhanced SDF-1α-induced chemotaxis. Short-term treatment with plerixafor inhibited SDF-1α-induced chemotaxis in a brief assay, but chemotaxis was enhanced by SDF-1α over a longer chemotaxis period. These findings suggest that these increases in surface CXCR4 are functional and that plerixafor-treated ALL blasts may have the potential to have increased interactions with the bone marrow microenvironment, highlighting a possible mechanism of resistance to CXCR4 inhibition. Therefore, the dosing of plerixafor (i.e., daily or constant exposure vs. intermittent or short-term exposure) is likely to impact its effectiveness as a chemosensitizing agent in ALL.

We and others have demonstrated that inhibition of CXCR4 enhances sensitivity to anti-leukemic therapy, and, CXCR4 inhibition has the potential to improve outcome in both high-risk ALL and AML. For example, we previously demonstrated that inhibition of CXCR4 with plerixafor enhances the cytotoxic effects of FLT3 inhibitors in *MLL-*R infant ALL[9] and chemotherapy in AML with internal tandem duplications of FLT3.[10] CXCR4 inhibition as a chemosensitization strategy has also moved into clinical trials. A phase 1/2 study that combined plerixafor with mitoxantrone, etoposide, and cytarabine in adults with relapsed or refractory AML demonstrated tolerability and a complete response (CR) rate of 39% and a combined CR and CR with incomplete blood count recovery (CRi) rate of 46%.[15] The preliminary results of two other clinical trials have been reported in abstract form. A phase 1 study in adults with newly-diagnosed AML tested the combination of plerixafor with the standard “7+3” regimen of cytarabine and daunorubicin. Preliminary results from 21 patients demonstrated a CR rate of 67% and a CR+CRi rate of 76%.[16] A phase 1 trial in children and young adults with relapsed or refractory ALL, AML, or myelodysplastic syndrome combined plerixafor with high-dose cytarabine and etoposide. The regimen was well-tolerated and there were no dose-limiting toxicities. The combined CR+CRi rate in this heterogeneous patient population was 16.7%.[17] All of these trials administered plerixafor for at least five consecutive days. While these results are promising for this strategy, the leukemic microenvironment is complex and has many other interactions that are of potential importance. We have shown that continuous CXCR4 inhibition with plerixafor may cause unintended effects on expression of molecules that mediate cell migration and adhesion, which may then influence how surviving blasts interact with the bone marrow microenvironment. It is possible that intensification of the anti-leukemic treatment backbone and/or the inclusion of additional microenvironment-targeted agents may overcome these potential shortcomings. Therefore, additional careful studies of CXCR4 inhibitors and other microenvironment-targeted agents must be performed in order to determine their optimal use in ALL.

## METHODS

### Cell culture

ALL cell lines (697, HB-1119, Nalm-6, RS4;11, and SEMK2) were purchased from ATCC and DSMZ. Cell lines were maintained in RPMI 1640 (Invitrogen, Carlsbad, CA) supplemented with 10% fetal bovine serum (FBS, Gemini Bio-Products, West Sacramento, CA), 1% 100x penicillin-streptomycin (Invitrogen), and 1% 200 mM L-glutamine (Invitrogen). Normal human bone marrow was collected from healthy adult bone marrow transplant donors according to a Johns Hopkins institutional review board-approved protocol. Stromal cells were isolated and maintained as previously described and plated into 96-well tissue culture plates for leukemia-stroma co-culture experiments.[9,10]

### Flow cytometry

Cells were stained with various antibodies (CXCR4 12G5-APC, eBioscience, San Diego, CA; CXCR4 12G5-PE-Cy5; CXCR4 1D9-PE; CXCR4 2B11-APC, eBioscience; CD49d-PE-Cy5; CXCR7-PerCP, R&D Systems, Minneapolis, MN) and read on a FACSCalibur (BD Biosciences, San Diego, CA). All antibodies were purchased from BD Biosciences unless otherwise noted. Results were analyzed using FlowJo (Tree Star, Inc., Ashland, OR). Gates were drawn around the live populations as determined by forward scatter and side scatter properties, and the geometric mean fluorescence index (MFI) of live cells was calculated. MFI results were normalized to the MFI of the corresponding isotype control.

### Apoptosis assays

Stock solutions of cytarabine (AraC), daunorubicin, and vincristine were prepared according to the manufacturer's instructions (Sigma-Aldrich, St. Louis, MO) and stored at −80°C until ready for use. Cell lines were pretreated with chemotherapy or vehicle control for 72 hours. After pretreatment, density centrifugation with Ficoll-Paque PLUS (GE Healthcare, Piscataway, NJ) was used to enrich for viable cells. Flow cytometry was performed using aliquots from each pretreatment condition to measure surface CXCR4 expression. Additional aliquots from each pretreatment condition were treated with dose ranges of chemotherapy or vehicle control for an additional 72 hours in three different treatment conditions: no stromal support, stromal co-culture, or treatment with plerixafor 5 μM (kindly provided by Genzyme, Cambridge, MA) for 30 minutes followed by stromal co-culture. The 96 well plate stroma cultures were washed 3 times with PBS prior to the addition of leukemia cells. Cells were then harvested and stained with Annexin V-PE and 7-AAD, evaluated by flow cytometry, and analyzed with FlowJo. Inhibitory concentration (IC) values were calculated using Calcusyn (Biosoft, Cambridge, U.K.) and used to calculate the Protective Index and Reversal Index.[10]

### Xenograft model: primary transplant, secondary transplant, treatment

Diagnostic bone marrow or peripheral blood samples were collected under institutional review board-approved protocols from newly diagnosed children with ALL. At the time of collection, mononuclear cells were isolated by Ficoll density centrifugation and red blood cell lysis. Mononuclear cells were then viably cryopreserved in 90% FBS with 10% DMSO and stored in liquid nitrogen. Vials of primary ALL cells were thawed and resuspended in RPMI, counted, and resuspended in cold PBS. Prior to transplant, the percentage of leukemic blasts in each sample (co-expressing human CD45 and human CD19) was determined by flow cytometry. Adult NOD/SCID/γ_c_^null^ mice (NSG, Jackson Laboratories, Bar Harbor, ME) were sublethally irradiated (200 centiGray) 4 hours prior to transplantation and 1×10^6^ primary ALL cells were transplanted via tail vein injection. All NSG mice were started on Uniprim medicated feed (Harlan Laboratories, Frederick, MD) at least 24 hours prior to transplantation to decrease opportunistic infections. Four to six weeks post-transplant, peripheral blood was collected via cheek venipuncture and analyzed by flow cytometry for the presence of leukemic blasts. The xenografts were sacrificed two to four weeks after successful engraftment and leukemic cells were harvested from the spleen and bone marrow: flow cytometry was performed at the time of harvest to quantify the percentage of leukemic blasts. Leukemic blasts (1×10^6^ per mouse) were then secondarily transplanted into sublethally irradiated NSG mice for treatment experiments. After a two-week period of engraftment, treatment was initiated as described above. After treatment, cells were isolated from the bone marrow, spleen, liver, and peripheral blood and flow cytometry was performed.

### Chemotaxis

Cells were treated with plerixafor or vehicle control, washed, resuspended in serum-free RPMI, and seeded into Millicell hanging cell culture inserts (8 μM pore size, Millipore, Billerica, MA). Chemotaxis toward serum-free media or serum-free media with human recombinant SDF-1α (150 ng/mL) (Peprotech, Rocky Hill, NJ) was measured as previously described.[10]

### Quantitative real-time PCR (qRT-PCR)

Measurement of mRNA expression of CXCR4, ITGA4, and CXCR7 was performed using TaqMan primers (Invitrogen) with glyceraldehyde-3-phosphate dehydrogenase (GAPDH) as a control as previously described.[10]

### Statistical analysis

P values were calculated using paired and independent two-sample t tests. Alpha was set to 0.05.

### Authorship

E.A.S, D.M., L.L., C.E.A., and R.E.R. performed the research; E.A.S. and P.B. designed the research; D.S. contributed mice for the study; E.A.S., D.M., C.E.A., R.E.R., and P.B. analyzed data; and E.A.S. and P.B. wrote the manuscript.

## SUPPLEMENTARY MATERIAL AND FIGURES


