# Supplementary material for: Plerixafor as a chemosensitizing agent in pediatric acute lymphoblastic leukemia: efficacy and potential mechanisms of resistance to CXCR4 inhibition
Source: Oncotarget. 2014 Aug 29;5(19):8947–58. doi: 10.18632/oncotarget.2407 (PMC4253409; doi:10.18632/oncotarget.2407)
Supplement: Supplementary file 1 [file oncotarget-05-8947-s001.pdf]

Plerixafor as a chemosensitizing agent in pediatric acute lymphoblastic leukemia: efficacy and potential mechanisms of resistance to CXCR4 inhibition

Supplementary Material

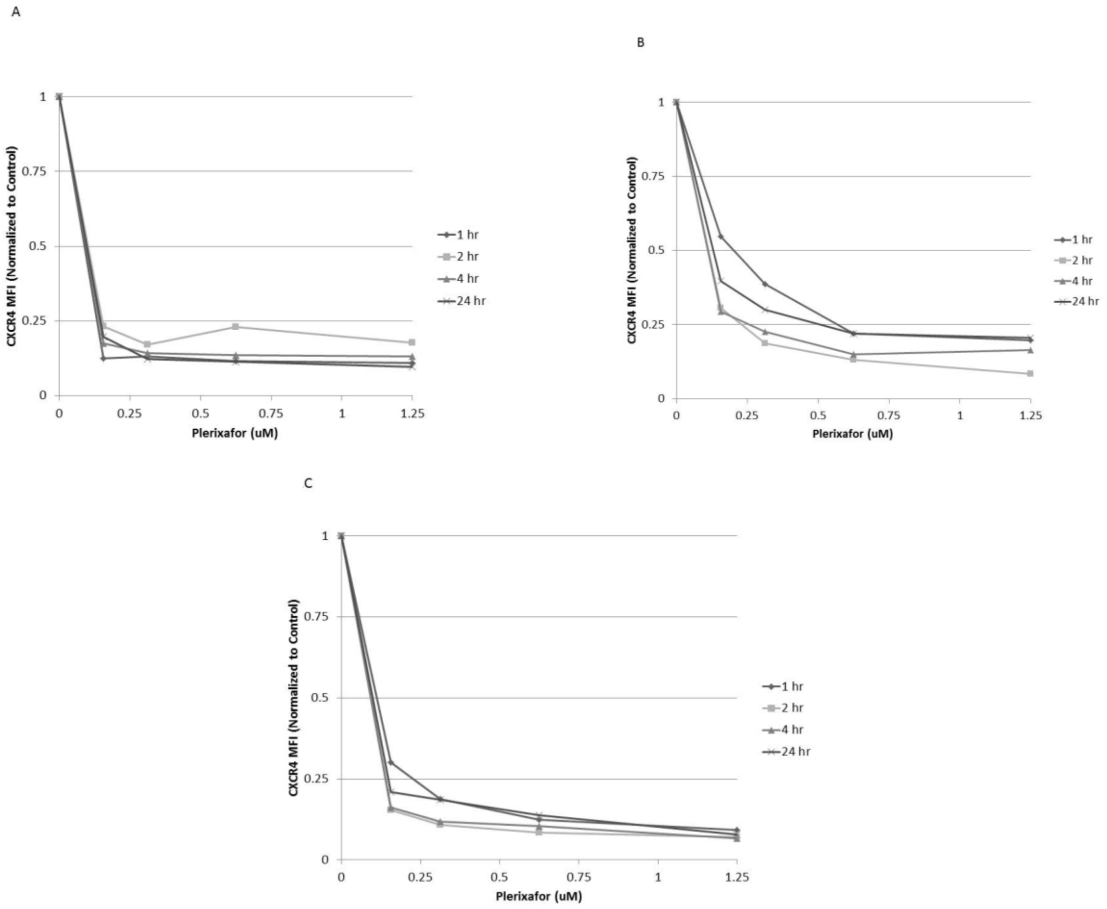

Supplemental Figure 1: Plerixafor decreases surface CXCR4 expression as measured by anti CXCR4 antibody binding Cell lines were treated with plerixafor over a time course. Surface CXCR4 as measured by 12G5 antibody binding in (A) 697, (B) HB-1119, and (C) RS4;11.

A

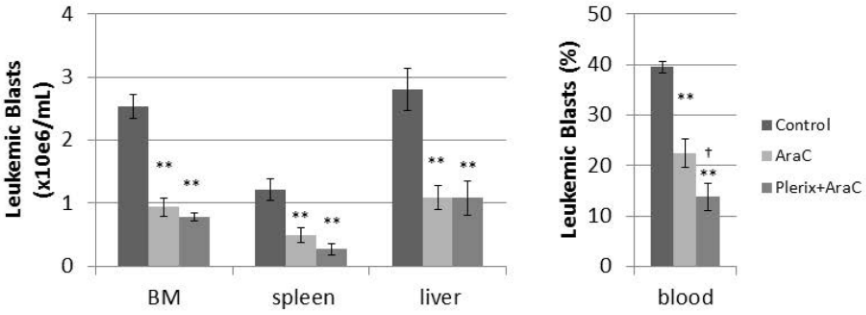
Leukemic Blasts (x10e6/mL)
4
3
2
1
0
**
**
**
**
**
**
BM
spleen
liver
Leukemic Blasts (%)
50
40
30
20
10
0
**
†
**
blood
Control
AraC
Plerix+AraC

B

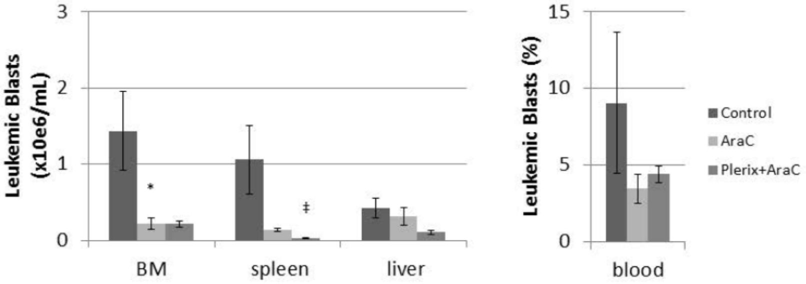
Leukemic Blasts (x10e6/mL)
3
2
1
0
*
‡
BM
spleen
liver
Leukemic Blasts (%)
15
10
5
0
blood
Control
AraC
Plerix+AraC

C

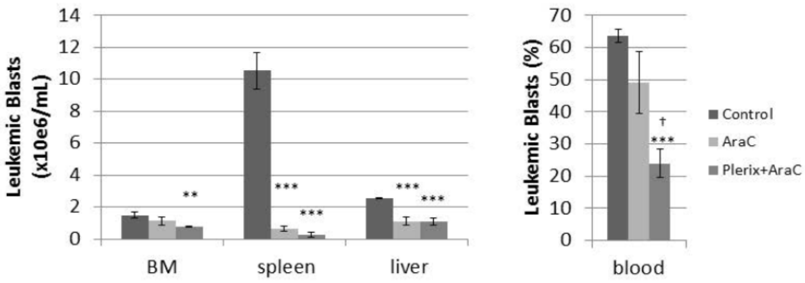
Leukemic Blasts (x10e6/mL)
14
12
10
8
6
4
2
0
**
***
***
***
***
BM
spleen
liver
Leukemic Blasts (%)
70
60
50
40
30
20
10
0
†
***
blood
Control
AraC
Plerix+AraC

D

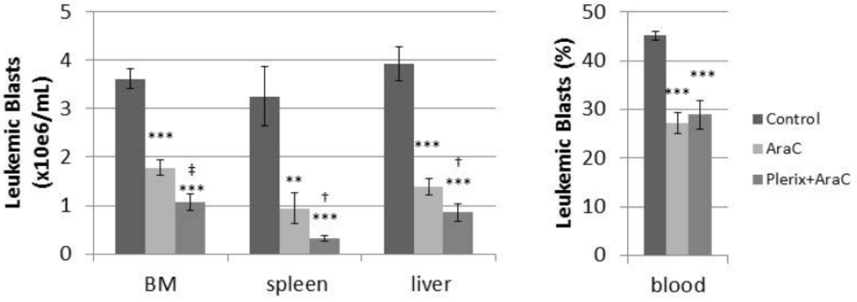

E

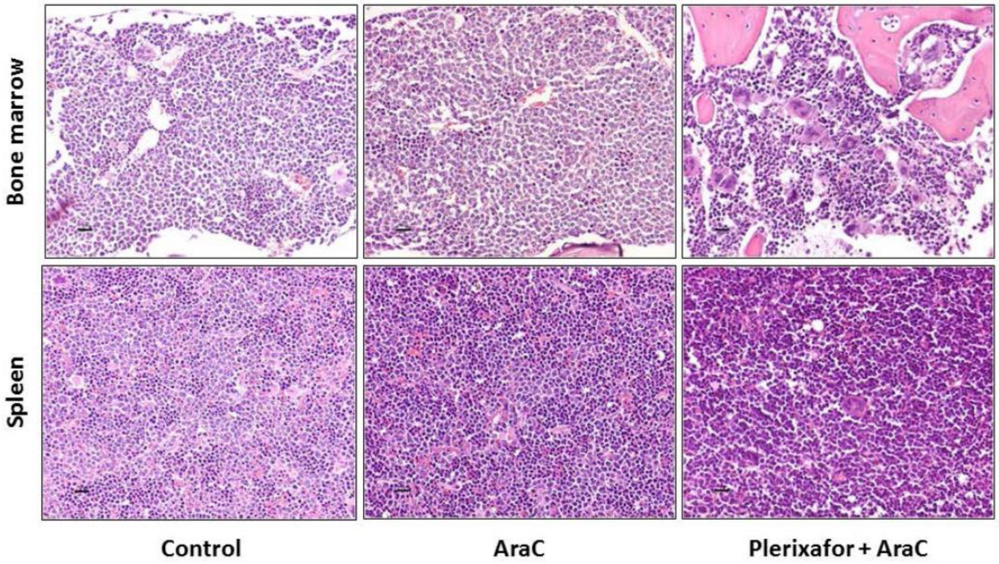

Supplemental Figure 2: In vivo treatment with plerixafor sensitizes leukemic blasts to AraC Leukemic mice were treated according to the treatment schema outlined in Figure 3C. Leukemic burden was quantified as the absolute blast count in each individual primary sample: (A) MLL1, (B) MLL2, (C) MLL3, and (D) MLL4. (E) Pathologic specimens from MLL1-transplanted mice. BM, bone marrow. *p<0.05, **p<0.01, ***p<0.001 vs. control. †p<0.05, ‡p<0.01 vs AraC.

A

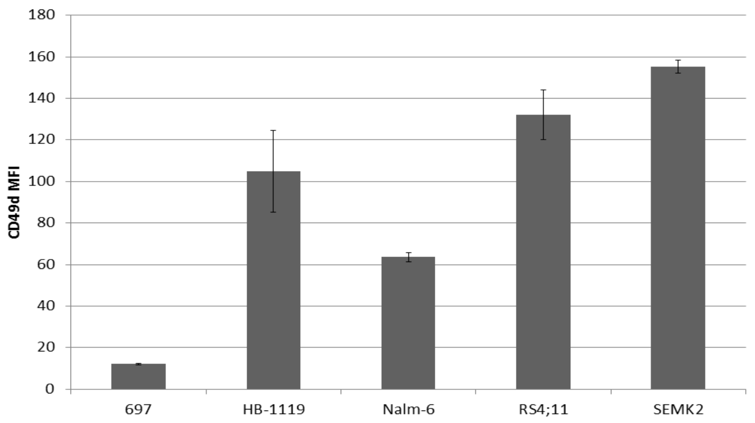

B

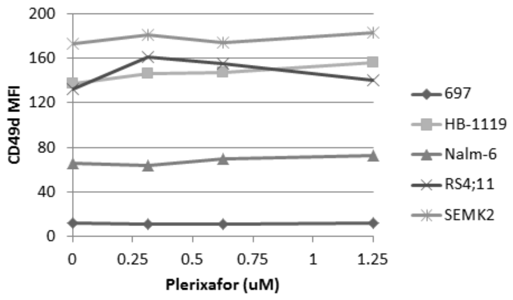

C

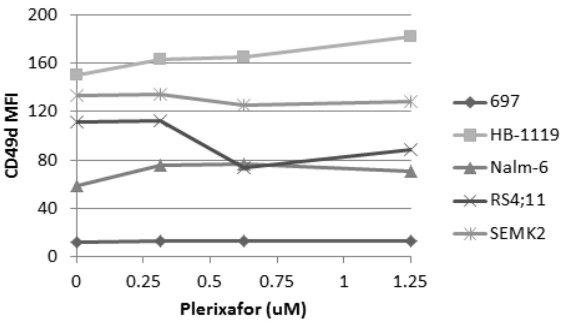

Supplemental Figure 3: Short-term treatment with plerixafor does not modulate surface CD49d expression (A) Baseline surface CD49d expression as measured by flow cytometry. Surface CD49d expression (B) 4 hours and (C) 24 hours after treatment with a dose range of plerixafor.

A

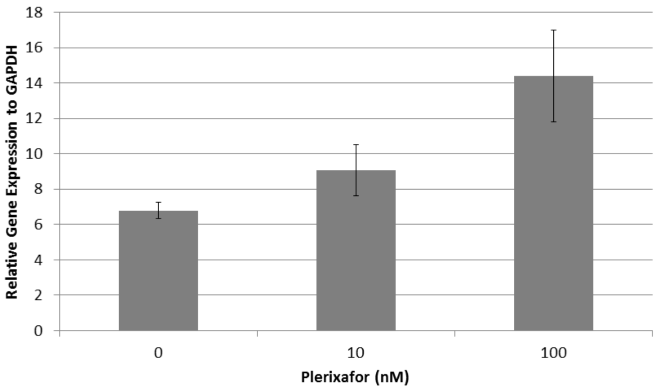

18
16
14
12
10
8
6
4
2
0
Relative Gene Expression to GAPDH
0
10
100
Plerixafor (nM)

B

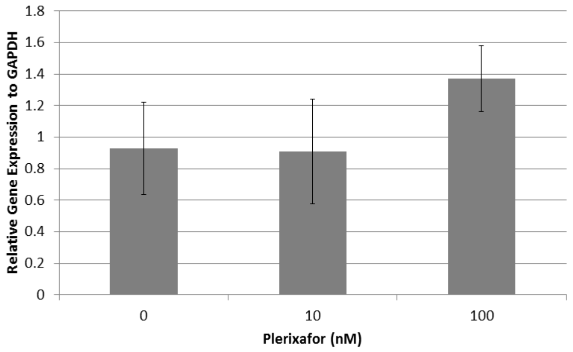

1.8
1.6
1.4
1.2
1
0.8
0.6
0.4
0.2
0
Relative Gene Expression to GAPDH
0
10
100
Plerixafor (nM)

C

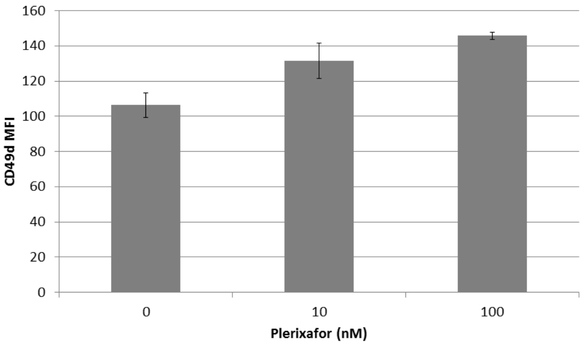

160
140
120
100
80
60
40
20
0
CD49d MFI
0
10
100
Plerixafor (nM)

D

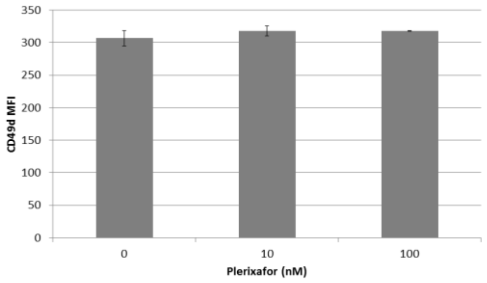

350
300
250
200
150
100
50
0
CD49d MFI
0
10
100
Plerixafor (nM)

E

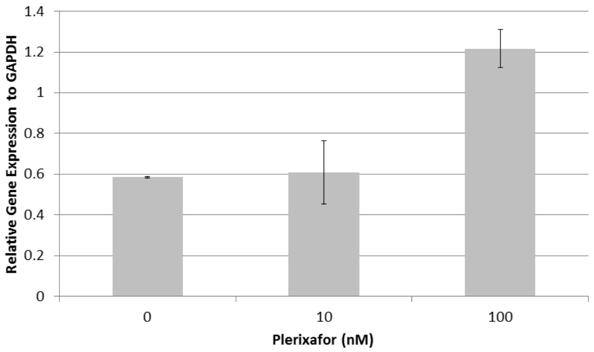

1.4
1.2
1
0.8
0.6
0.4
0.2
0
Relative Gene Expression to GAPDH
0
10
100
Plerixafor (nM)

F

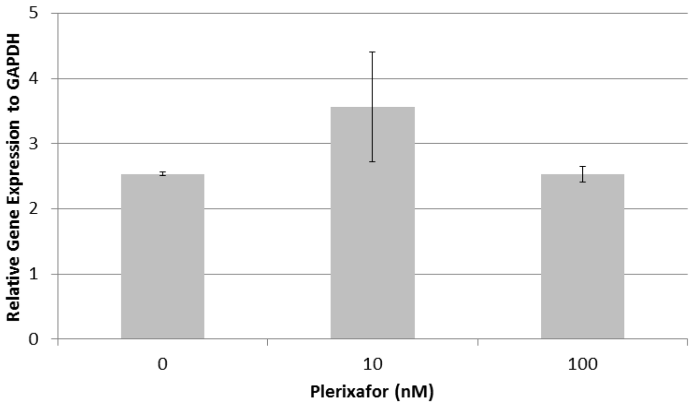

5
4
3
2
1
0
Relative Gene Expression to GAPDH
0
10
100
Plerixafor (nM)

G

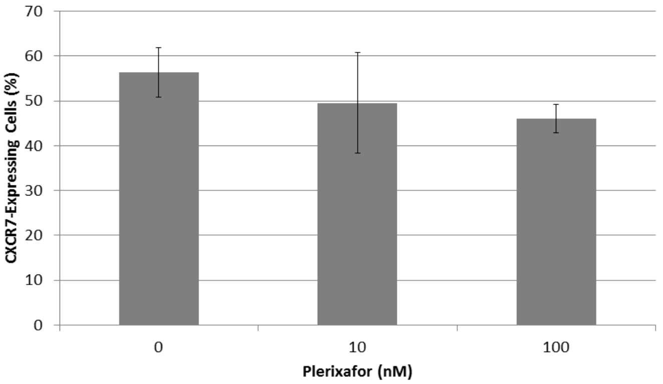

70
60
50
40
30
20
10
0
CXCR7-Expressing Cells (%)
0
10
100
Plerixafor (nM)

H

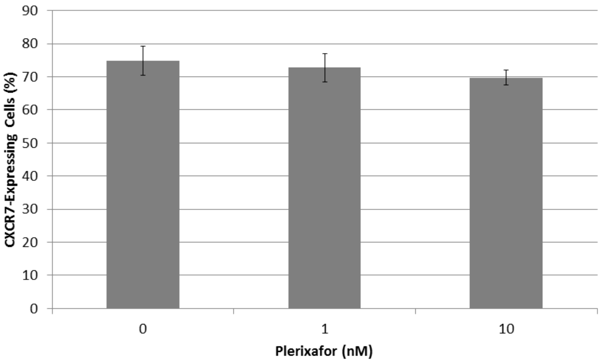

90
80
70
60
50
40
30
20
10
0
CXCR7-Expressing Cells (%)
0
1
10
Plerixafor (nM)

I

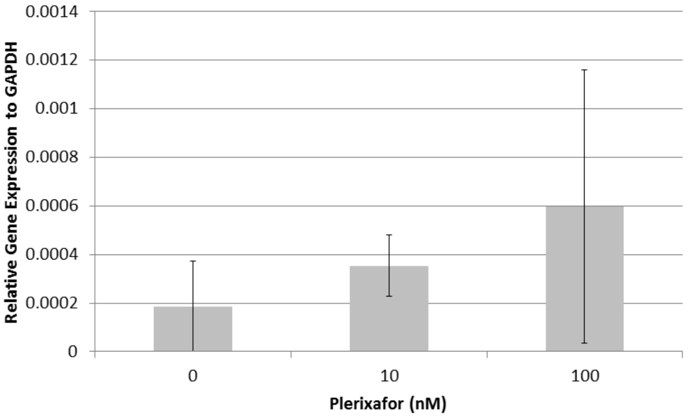

0.0014
0.0012
0.001
0.0008
0.0006
0.0004
0.0002
0
Relative Gene Expression to GAPDH
0
10
100
Plerixafor (nM)

J

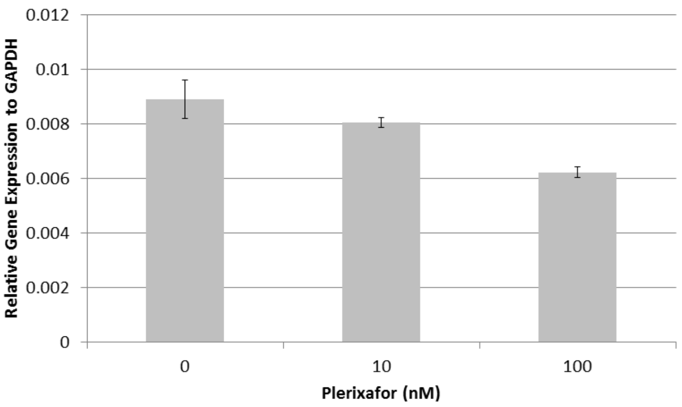

Supplemental Figure 4: Extended treatment with plerixafor modulates surface expression of adhesion molecules Cell lines were treated with plerixafor or vehicle control for 72 hours. CXCR4 expression by qRT-PCR in (A) Nalm-6 and (B) HB-1119. Surface expression of CD49d by flow cytometry in (C) Nalm-6 and (D) HB-1119. ITGA4 expression by qRT-PCR in (E) Nalm-6 and (F) HB-1119. Surface expression of CXCR7 by flow cytometry in (G) Nalm-6 and (H) HB-1119. CXCR7 expression by qRT-PCR in (I) Nalm-6 and (J) HB-1119.

A

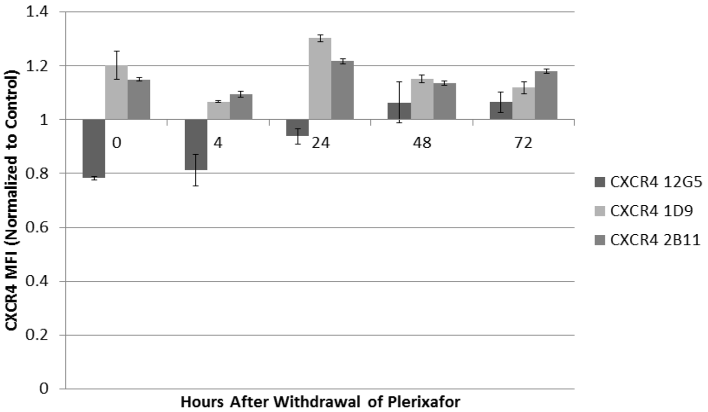

B

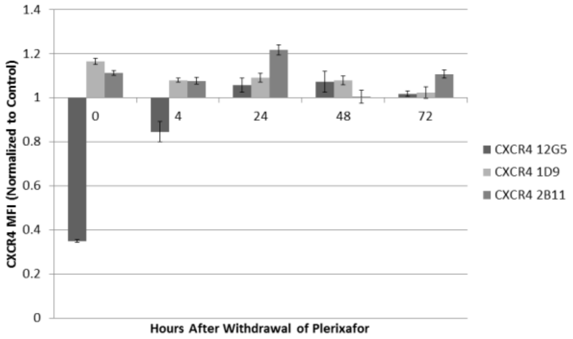

C

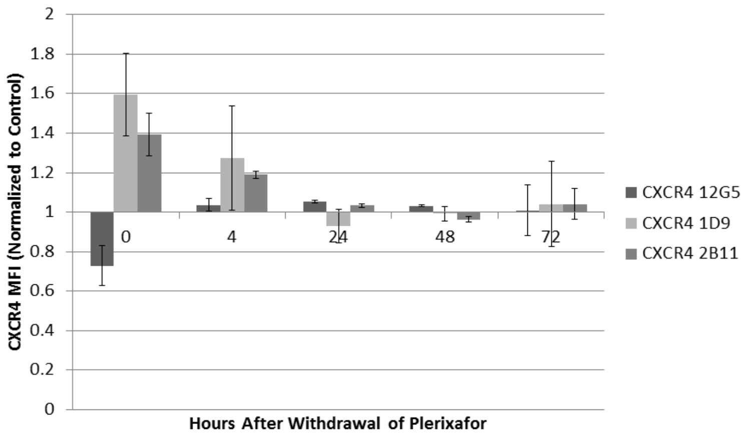

D

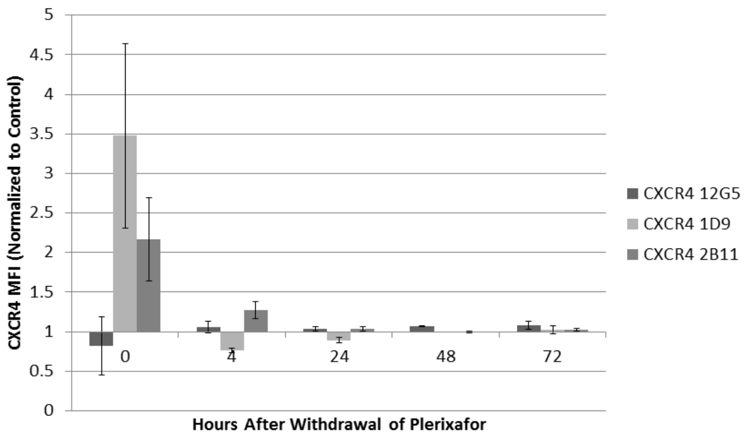

Supplemental Figure 5: The effects of extended treatment with plerixafor on surface CXCR4 expression persist over time Cell lines were treated with plerixafor or vehicle control for 72 hours. Cells were then washed and resuspended in fresh medium. Following withdrawal of plerixafor, cells were harvested for flow cytometry at various time points for an additional 72 hours. Surface expression of CXCR4 was measured using 12G5, 1D9, and 2B11 antibodies. Results for (A) Nalm-6 10 nM, (B) Nalm-6 100 nM, (C) HB-1119 10 nM, and (D) HB-1119 100 nM.
